# The Role of Preoperative Computed Tomography Radiomics in Distinguishing Benign and Malignant Tumors of the Parotid Gland

**DOI:** 10.3389/fonc.2021.634452

**Published:** 2021-03-10

**Authors:** Yuyun Xu, Zhenyu Shu, Ge Song, Yijun Liu, Peipei Pang, Xuehua Wen, Xiangyang Gong

**Affiliations:** ^1^ Department of Radiology, Zhejiang Provincial People’s Hospital, Affiliated People’s Hospital of Hangzhou Medical College, Hangzhou, China; ^2^ Department of Radiology, Zhejiang Cancer Hospital, Hangzhou, China; ^3^ Department of Pharmaceuticals Diagnosis, GE Healthcare, Hangzhou, China; ^4^ Institute of Artificial Intelligence and Remote Imaging, Hangzhou Medical College, Hangzhou, China

**Keywords:** CT, malignancy, parotid gland, radiomics, tumor

## Abstract

**Objective:**

This study aimed to develop and validate an integrated prediction model based on clinicoradiological data and computed tomography (CT)-radiomics for differentiating between benign and malignant parotid gland (PG) tumors *via* multicentre cohorts.

**Materials and Methods:**

A cohort of 87 PG tumor patients from hospital #1 who were diagnosed between January 2017 and January 2020 were used for prediction model training. A total of 378 radiomic features were extracted from a single tumor region of interest (ROI) of each patient on each phase of CT images. Imaging features were extracted from plain CT and contrast-enhanced CT (CECT) images. After dimensionality reduction, a radiomics signature was constructed. A combination model was constructed by incorporating the rad-score and CT radiological features. An independent group of 38 patients from hospital #2 was used to validate the prediction models. The model performances were evaluated by receiver operating characteristic (ROC) curve analysis, and decision curve analysis (DCA) was used to evaluate the clinical effectiveness of the models. The radiomics signature model was constructed and the rad-score was calculated based on selected imaging features from plain CT and CECT images.

**Results:**

Analysis of variance and multivariable logistic regression analysis showed that location, lymph node metastases, and rad-score were independent predictors of tumor malignant status. The ROC curves showed that the accuracy of the support vector machine (SVM)-based prediction model, radiomics signature, location and lymph node status in the training set was 0.854, 0.772, 0.679, and 0.632, respectively; specificity was 0.869, 0.878, 0.734, and 0.773; and sensitivity was 0.731, 0.808, 0.723, and 0.742. In the test set, the accuracy was 0.835, 0.771, 0.653, and 0.608, respectively; the specificity was 0.741, 0.889, 0.852, and 0.812; and the sensitivity was 0.818, 0.790, 0.731, and 0.716.

**Conclusions:**

The combination model based on the radiomics signature and CT radiological features is capable of evaluating the malignancy of PG tumors and can help clinicians guide clinical tumor management.

## Introduction

### Background

Parotid gland (PG) tumors are rare and account for approximately 1%–3% of all head and neck tumors (1). Parotid tumors are a clinically, morphologically, radiologically diverse group of neoplasms that may present significant diagnostic and management challenges. Radical tumor resection with lymph node dissection remains the mainstay treatment for malignant parotid tumors, followed by adjuvant chemotherapy and radiotherapy (2). Knowledge of the clinical information and imaging characteristics before surgery would be of outstanding importance for evaluating these tumors, tailoring treatment decisions and optimizing individualized surgical plans. Additionally, for malignancies, preoperative knowledge of the tumor type would also be of paramount importance.

Currently, multiple imaging techniques are available to study the parotid region, such as ultrasound, computed tomography (CT), and magnetic resonance imaging (MRI). Although CT is not a first-line method for parotid gland tumor evaluation, it can be used to help clinicians evaluate PG tumors to confirm the presence of a parotid mass, assess the extent of tumor especially in the deep lobe, and detect enlarged lymph nodes, to facilitate the determination of benign or malignant nature of the tumor for appropriate treatment. However, CT involves radiation, and various neoplasms may have similar imaging features on CT (3, 4). Furthermore, contrast-enhanced CT may cause contrast-induced adverse reactions, though it is generally considered safe, with an overall prevalence of adverse reactions around 0.7%, among which most of the events (more than 80%) are mild (5). Ultrasound is a cheap and effective tool for delineating cystic versus solid tumors, tumor borders, and cervical lymph nodes; however, it poorly visualizes the deep lobe and is dependent on operator expertise. MRI has sufficiently high resolution to detect and evaluate parotid tumors noninvasively. As a functional MRI technique, DWI can be used to explore the diffusion changes in composition of tissues, which is helpful for parotid tumor detection and differential diagnosis (6). Nevertheless, its disadvantages include limited availability for patients with metal prostheses, high cost and long waiting times. Fine-needle aspiration biopsy (FNAB) is an accurate method for identifying the nature of these tumors; however, FNAB is invasive and may cause hemorrhage, facial nerve injury, and acute sialadenitis at the needle puncture site (7, 8). In addition, there are significant variations in the performance of FNAB within different practice settings which is associated with inadequate diagnoses and missed malignancies, with a sensitivity for detecting malignancy between 70% and 80% and non-diagnostic rates average at 14%–18% (9).Thus, challenges remain in non-invasively and accurately distinguishing benign from malignant lesions on pre-operative CT images.

Radiomics is new method of mining objective and quantitative features such as the shape, intensity, and energy of regions of interest from medical images (e.g., gray-level co-occurrence matrix (GLCM) and run length matrix (RLM) features), describing the relationships between image voxels far beyond the traditional visual features we can obtain and thus reflecting the underlying genetic and biological variability of the analyzed tissue, which can promote accurate diagnosis and individualize cancer treatment (10). Recent studies describe the use of radiomic analysis for head and neck tumors (11), glioblastoma (12), breast cancer (13), rectal cancer (14), hepatocellular carcinoma (15), etc. These studies demonstrated that radiomic features are closely associated with the histopathological types, grading and prognosis of tumors and can help solve many clinical problems and optimize patient treatment. A previous study (16) used energy spectrum CT, which is not commonly used, in the tissue classification of benign parotid tumors and demonstrated good discrimination ability.

However, as mentioned above, it is difficult to distinguish benign from malignant PG tumors with conventional CT images, but considering the novelty of radiomics and the powerful performance of tumor differential diagnosis in other field, we hypothesize that radiomics analysis based on conventional CT images can be used to distinguish between benign and malignant PG tumors. The purpose of this study is to utilize conventional CT images to extract PG tumor-related radiomic features and combine them with conventional radiological features to build a classification model for distinguishing benign and malignant PG tumors.

## Material and Methods

This retrospective study was approved by Ethics Committee of Zhejiang Provincial People’s Hospital. All patients’ informed consents were waived for the retrospective nature of this study.

The research method was carried out in accordance with the relevant guidelines and regulations. Patients’ clinical and image data were obtained from routine clinical records and the picture archiving and communication systems (PACS) of the hospital.

### Patient Population

**Figure 1:**
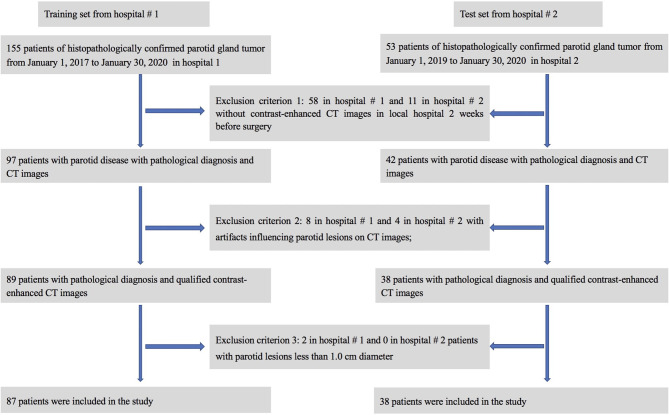
Flow chart for patient selection in this study.

A retrospective review of clinical and radiological databases was performed from January 1, 2017, to January 30, 2020, in hospital #1 and from Jan 1, 2019, to January 30, 2020, in hospital #2. The inclusion criteria were as follows: (1) patients with PG-related symptoms or masses; (2) confirmation of the PG tumor by surgery and postoperative pathological diagnosis; and (3) non-contrast computed tomography (NCCT) and contrast enhanced computed tomography (CECT) images of the head and neck containing the PG obtained within 2 weeks before the operation. The exclusion criteria were as follows: (1) CECT images with obvious artifacts, such as artifacts from false teeth, motion artifacts, etc.; (2) fine needle aspiration performed before imaging of the PG; or (3) patients with parotid lesions less than 1.0 cm in diameter. Medical records from 208 patients were initially analyzed, and 125 patients were finally included in this study (see **Figure 1** for details). In order to know whether there is selection bias, a comparison between the included and excluded dataset were performed.

CT scans were performed with a multi-slice CT scanner (Siemens 40) or a 64 multidetector scanners (LightSpeed VCT; GE Healthcare, Waukesha, WI, USA) with the following parameters: tube voltage of 120 kVp; tube current of 150 mA; section thickness of 3 mm; and section interval of 3 mm. The scanning ranged from the base of the skull to the inlet of the thorax. The CECT images were obtained after an intravenous injection of 80–100 ml of nonionic iodinated iopamidol containing 370 mg iodine per ml (Isovue 370, Bracco Healthcare, Princeton, NJ) at 3–4 ml/s. Arterial-phase CECT images were obtained 35 seconds after contrast material injection.

### Clinical and Radiological Data Analysis

Clinical parameters, including age, sex, disease duration, and smoking status, were collected from the hospital medical record system. All original CT images were reviewed and assessed by two experienced head and neck radiologists who were blinded to the clinical data, including tumor location (in the deep or shallow parotid), maximum diameter, distribution (single or bilateral), shape (round or not), capsule (with or without), regularity (regular or irregular), margin (clear or unclear), density (hypo-, iso-, or hyperdense), enhancement (enhanced or non-enhanced), cystic degeneration (with or without), lymph node metastasis (with or without), hemorrhage (with or without), calcification (with or without), and enhancement type (slight-moderate or obvious). The definition of some radiological features can be found in the **Supplementary Material**. Discordant interobserver interpretations were resolved by consensus. If there were multiple lesions in the parotid gland, the largest lesion with confirmed pathology was chosen for the analysis.

### Image Preprocessing

All NCCT and CECT images were stored in Digital Imaging and Communications in Medicine (DICOM) format and imported to ITK-SNAP software for three-dimensional manual segmentation of the region of interest (ROI). The ROI of each case was manually drawn on the CECT images by two independent head & neck radiologists (Radiologist X with 11 years of experience and Radiologist W with 6 years of experience) who were blinded to the clinical information, carefully avoiding the vessels, bones and lymph nodes. All ROIs were then replicated to the NCCT images, and manual correction was also performed to adjust small deviations in delineating the ROI boundaries. All ROIs from the NCCT and CECT images were uploaded into AK analysis software (Artificial Intelligence Kit V3.0.0. R, GE Healthcare) for feature extraction. To eliminate the potential impact of different imaging parameters on the extracted features, we preprocessed the segmented images, including resampling the images to 1×1 ×1 mm^3^ voxel size, intensity normalizing, and standardizing the gray levels to range from 1 to 32 (17).

### Radiomics Feature Selection

The preprocessed images were used to extract the radiomics features, including the histogram, Haralick, FormFactor, gray level co-occurrence matrix (GLCM), run length matrix (RLM) and gray level size zone matrix (GLZSM) features. In this study, a joint feature set was obtained from both the NCCT and CECT images. The most robust features of the two separate ROI datasets from the two radiologists were used to ensure the reproducibility and repeatability of the radiomics features (18). Spearman’s rank test was utilized to evaluate the correlation coefficients between the features of the datasets segmented by Radiologist X and Radiologist W. Any features that had correlation coefficients greater than 0.8 were defined as “robust” features (19). A large quantity of features with a limited sample size may hinder the predictive ability of the model, especially in a high-dimensional feature space, owing to the “curse of dimensions” (20)^;^ therefore, the dimensions of the extracted features were reduced to address this issue. Analysis of variance was first performed on the extracted features to select those features that were statistically significant. Subsequently, the minimum redundancy maximum correlation (mRMR) algorithm was used to reduce the dimensions of the selected features as well as to select the features that had the highest correlation with the tumor classification and had the smallest redundancy between one another. After that, the emerging gradient boosting decision tree (GBDT) algorithm was used to further reduce the dimensionality of the preselected features. In this study, feature selections were performed on both the NCCT and CECT images of all cases and finally obtained a joint feature set containing NCCT and CECT image features. Based on these selected features, logistic regression was used to construct the radiomics signature.

To determine the correlation between the radiomics signature and tumor classification, a logistic regression (LR)-based signature model based on the combined feature set was used to calculate a score to reflect the actual tumor classification in the training set, defined as the rad-score, which was then used to determine the effectiveness of the signature models in differentiating between patients with benign and malignant parotid tumors. The formula of the model used in the training set was then employed to calculate the scores for the test set.

The area under the curve (AUC) of the receiver operating characteristic (ROC) curve was used to evaluate the accuracy of the radiomics signature in both the training and validation sets. The calibration performance was assessed with the calibration curve for the continuous variables. Furthermore, decision curve analysis (DCA) was used to assess the clinical efficiency of the radiomics signature in classifying the tumor by calculating the net benefit.

### Prediction Model Construction and Validation

**Figure 2:**
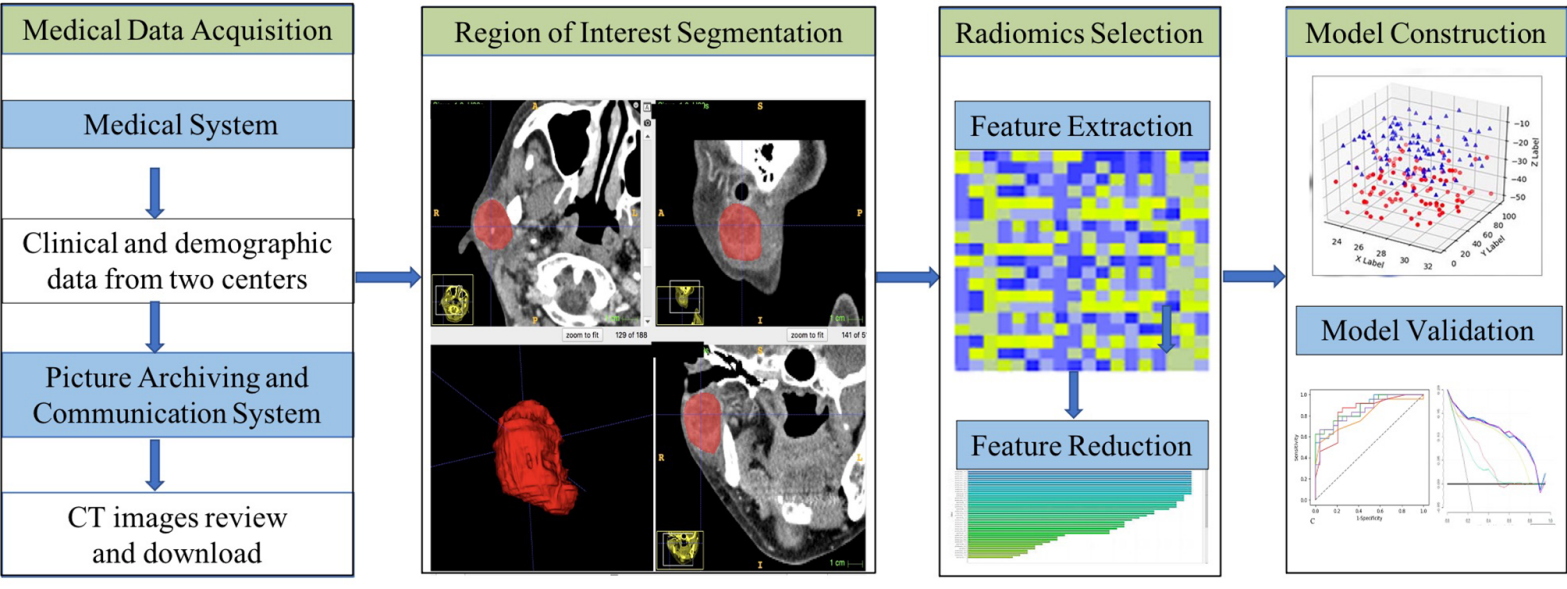
Workflow of this study.

One-way analysis of variance was performed for each potential predictor variable including clinical characteristics, radiological characteristics, and the radiomics signature in the training group, and then multivariable logistic regression was used on the preselected features with significant differences to obtain the predictors that were ultimately employed for model construction. As machine learning can provide highly accurate and reliable models to improve clinical oncology decisions, in this study, we chose a support vector machine (SVM) to build a combined prediction model based on the selected predictors. The performance of the model was validated with the training set and the test set separately, including calibration performance assessed with the calibration curve, diagnostic accuracy using ROC analysis and net benefit evaluated by DCA. In addition, the tumor prediction value of each patient in the training set and test set was calculated according to the model, the cut-off value of the ROC curve was used to divide parotid tumors into low-risk and high-risk groups, and the clinical effectiveness was determined by the actual tumor classification in the different groups. **Figure 2** shows the workflow of this study.

### Statistics Analysis

SPSS 17.0 software (IBM, Chicago, IL, USA) was used to evaluate the normality of the distribution of the dataset using the Kolmogorov-Smirnov test and to perform the chi-square test for the categorical data. The T test is used for normally distributed variables, and the Mann-Whitney test is used for non-normally distributed variables. The conditional forward stepwise selection method was applied for the multivariable logistic regression model. MedCalc15.8 software (MedCalc, Ostend, Belgium) was used to assess the ROC curves for the diagnostic performance of the models, and differences between the various AUCs were compared with the DeLong test. R statistical software was used for all other statistical analyses. The “mRMRe” and “gbm” packages were used for mRMR and GBDT analyses, respectively. DCA plots were generated with the “dca. R” package. Two-tailed p-values less than 0.05 were considered statistically significant.

## Results

### Clinical Features

**Figure 3:**
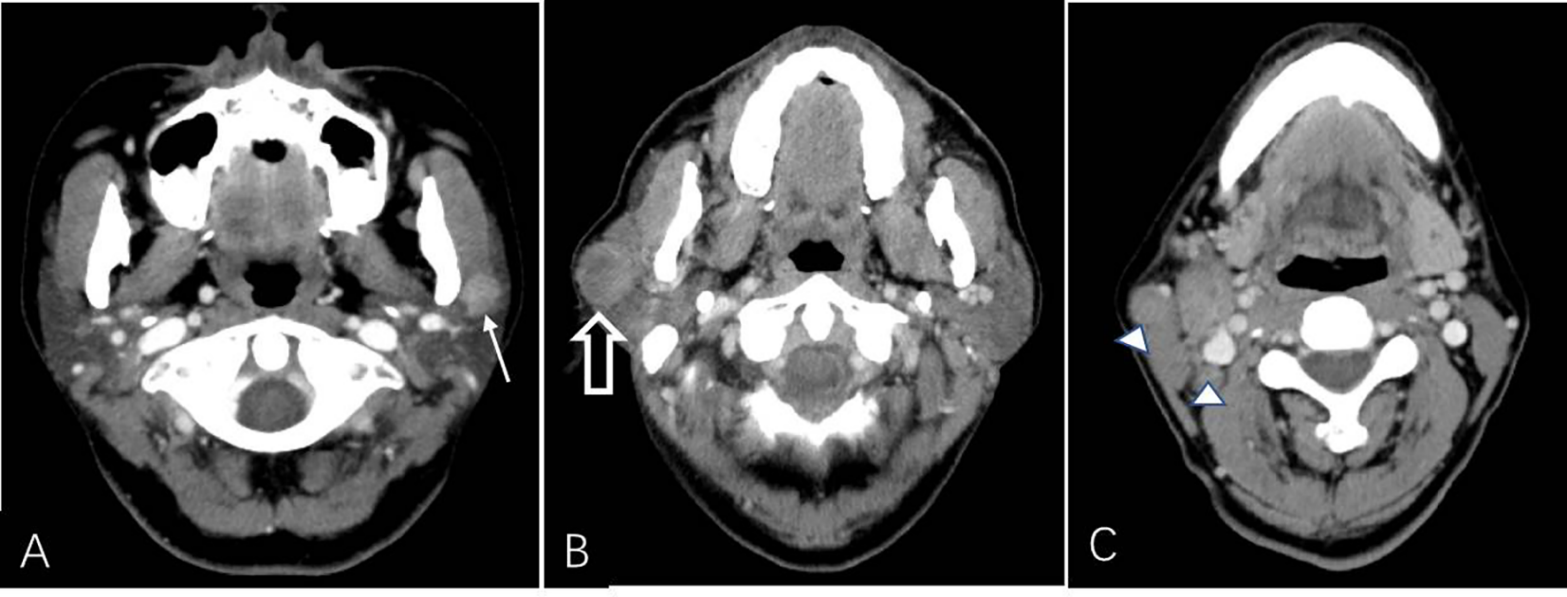
CT example images of benign and malignant parotid tumors. **(A)** Axial plain CT image of a 60-year-old male patient without a smoking history presenting with a left neck mass (solid white arrow). The patient had undergone parotidectomy 3 days after CT examination, and the mass was histopathologically confirmed as a Warthin tumor. **(B, C)** Axial contrast-enhanced CT image of a 59-year-old male patient with a 20-year smoking history presenting with a right neck mass (open white arrow, **B**) that was surgically removed and histopathologically confirmed as a salivary ductal carcinoma with lymph node metastasis (△, **C**). The mass shows heterogeneous enhancement with an irregular and unclear border.

A total of 125 patients (63 male and 62 female) with PG tumors were included from two medical centers. The results showed that there was no significant difference between the included and excluded datasets (see **Table 1** in the **Supplementary Materials** for details). Seventeen different tumor types were represented, most of which were benign tumors, especially pleomorphic adenomas and Warthin tumors. The details of the PG tumors are shown in **Table 1**. The clinical and radiological features of the patients in the training set and test set are shown in **Table 2**. No statistically significant difference was found for any of the data between the two groups. In the training set, the tumor location and borders and lymph node status were significantly different between benign and malignant PG tumors. In the test group, there were significant differences only in tumor location (**Table 3**). **Figure 3** shows an example of radiological feature analysis of two cases.

**Table 1:** Details of the parotid tumors.

Parotid gland tumor	Number
Pleomorphic adenoma	53
Warthin tumor	25
Squamous cell carcinoma	10
Lymphoma	9
Salivary ductal carcinoma	5
Acinic cell carcinoma	4
Mucoepidermoid carcinoma	4
Basal cell adenoma	3
Myoepithelial tumor	2
Haemangioma	2
Lymphoepithelial carcinoma	2
Malignant pleomorphic adenoma	1
Basal cell adenoma	1
Mammary analog secretory carcinoma	1
Schwannoma	1
Oncocytoma	1
Adenoid cystic carcinoma	1
Total	125

**Table 2:** Clinical and radiological characteristics of patients in the training and test sets.

Variable		Training set (n=87)	Test set (n = 38)	*P* value
Age (years)		52.6 ± 16.8	55 ± 14.8	0.446
Duration (months)		31.8 ± 62.3	21.3 ± 48.3	0.356
Maximum diameter (cm)		26 ± 11.9	29.3 ± 22.1	0.385
Sex [n (%)]	Male	39(44.8)	24(63.2)	0.059
	Female	48(55.2)	14(36.8)	
Smoking [n (%)]	Yes	18(20.7)	13(34.2)	0.107
	No	69(79.3)	25(65.8)	
Distribution	Single	76(87.4)	35(92.1)	0.439
	Bilateral	11(12.6)	3(7.9)	
Location	Deep	15(17.2)	9(23.7)	0.4
	Shallow	72(82.8)	29(76.3)	
Shape	Round	15(17.2)	10(26.3)	0.4
	Non-rounded	72(82.8)	28(73.7)	
Capsule	Yes	40(46)	17(44.7)	0.898
	No	47(54)	21(55.3)	
Regularity	Yes	40(46)	23(60.5)	0.135
	No	47(54)	15(39.5)	
Border	Clear	47(54)	24(63.2)	0.343
	Unclear	40(46)	14(36.8)	
Density	Hypodense	36(41.4)	15(39.5)	0.842
	Iso-hyperdense	51(58.6)	23(60.5)	
Enhancement	Yes	79(90.8)	36(94.7)	0.456
	No	8(9.2)	2(5.3)	
Marked Enhancement	Yes	60(69)	24(63.2)	0.525
	No	27(31)	14(36.8)	
Cystic degeneration	Yes	39(44.8)	18(47.4)	0.793
	No	48(55.2)	20(52.6)	
Lymphadenopathy	Yes	14(16.1)	8(21.1)	0.503
	No	73(83.9)	30(78.9)	
Hemorrhage	Yes	1(1.1)	1(2.6)	0.544
	No	86(98.9)	37(97.4)	
Calcification	Yes	4(4.6)	1(2.6)	0.606
	No	83(95.4)	37(97.4)	
Heterogeneity	Yes	32(36.8)	14(36.8)	0.995
	No	55(63.2)	24(63.2)	

**Table 3:** Clinical and radiological characteristics between benign and malignant parotid gland tumors in the training and test sets.

Variable		Training set (n=87)			Test set (n = 38)		
		Benign (n=61)	Malignant (n =26)	*P* value	Benign (n=27)	Malignant (n =11)	*P* value
Age (years)		52.2 ± 17.5	53.4 ± 15.4	0.781	53.9 ± 16.4	57.6 ± 10.3	0.496
Duration (months)		31.3 ± 59.2	33.2 ± 70.5	0.893	25.2 ± 56.3	11.7 ± 15.7	0.44
Maximum diameter (cm)		25 ± 11.9	28.3 ± 11.8	0.234	27.3 ± 22.7	34.5 ± 20.6	0.37
Sex [n (%)]	Male	30 (49.2)	9 (34.6)	0.211	18 (66.7)	6 (54.5)	0.482
	Female	31(50.8)	17 (65.4)		9(33.3)	5(45.5)	
Smoking [n (%)]	Yes	15 (24.6)	3 (11.5)	0.169	10 (37)	3(27.3)	0.565
	No	46(75.4)	23 (88.5)		17 (63)	8 (72.7)	
Distribution	Single	52(85.2)	24 (92.3)	0.364	25(92.6)	10 (90.9)	0.861
	Bilateral	9 (14.8)	2 (7.7)		2 (7.4)	1 (9.1)	
Location	Deep	4(6.6)	11 (42.3)	<0.001*	4(14.8)	5 (45.5)	0.044*
	Shallow	57 (93.4)	15 (57.7)		23 (85.2)	6(54.5)	
Shape	Round	10(16.4)	5 (19.2)	0.748	8(29.6)	2 (18.2)	0.467
	Non-rounded	51 (83.6)	21 80.8)		19 (70.4)	9 (81.8)	
Capsule	Yes	32 (52.5)	8 (30.8)	0.063	13 (48.1)	4 (36.4)	0.508
	No	29(47.5)	18 (69.2)		14(51.9)	7 (63.6)	
Regularity	Yes	31(50.8)	9 (34.6)	0.165	17(63)	6 (54.5)	0.63
	No	30 (49.2)	17(65.4)		10 (37)	5 (45.5)	
Border	Clear	38(62.3)	9 (34.6)	0.018*	19(70.4)	5 (45.5)	0.149
	Unclear	23 (37.7)	17(65.4)		8 (29.6)	6 (54.5)	
Density	Hypodense	26(42.6)	10 (38.5)	0.718	11(40.7)	4 (36.4)	0.802
	Iso-hyperdense	35 (57.4)	16 61.5)		16(59.3)	7(63.6)	
Enhancement	Yes	55 (90.2)	24 (92.3)	0.751	25 (92.6)	11 (100)	0.354
	No	6(9.8)	2 (7.7)		2(7.4)	0 (0)	
Marked Enhancement	Yes	40 (65.6)	20 (76.9)	0.295	17 (63)	7 (63.6)	0.969
	No	21(34.4)	6 (23.1)		10(37)	4 (36.4)	
Cystic degeneration	Yes	28 (45.9)	11 (42.3)	0.758	12 (44.4)	6 (54.5)	0.572
	No	33(54.1)	15 (57.7)		15(55.6)	5 (45.5)	
Lymphadenopathy	Yes	5 (8.2)	9 (34.6)	0.002*	4 (14.8)	4 (36.4)	0.139
	No	56(91.8)	17 (65.4)		23(85.2)	7 (63.6)	
Hemorrhage	Yes	0 (0)	1 (3.8)	0.123	1 (3.7)	0 (0)	0.518
	No	61(100)	25 96.2)		26(96.3)	11 (100)	
Calcification	Yes	2 (3.3)	2 (7.7)	0.368	1 (3.7)	0 (0)	0.518
	No	59(96.7)	24 92.3)		26(96.3)	11 (100)	
Heterogeneity	Yes	24(39.3)	8 (30.8)	0.448	12(44.4)	2 (18.2)	0.128
	No	37 (60.7)	18(69.2)		15 (55.6)	9 (81.8)	

*represents P < 0.05.

### Radiomics Signature Construction and Validation

**Figure 4:**
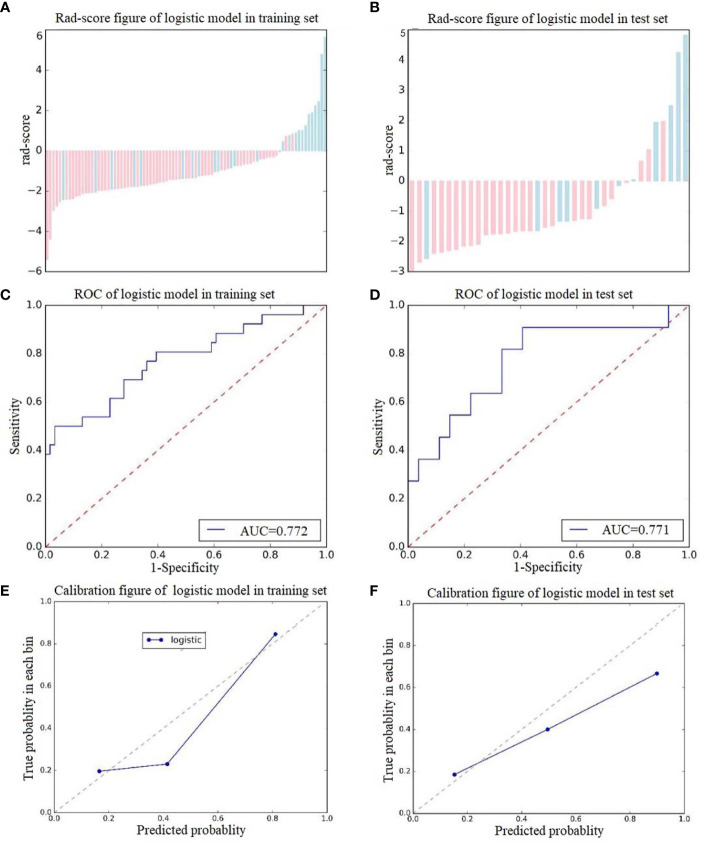
Score diagrams of the radiomics signature in **(A)** the training set and **(B)** the test set. **(C, D)** show the diagnostic accuracy of the rad-score of the radiomics signature in the training and test groups. **(E, F)** show the calibration curve of the radiomics signature in the training and test sets.

A total of 378 radiomics features were extracted from a single ROI; thus, 756 radiomics features were extracted from each patient in the two scan phases. Among these features, 14 retained after feature dimensionality reduction, including 5 features from NCCT images (HaralickCorrelation_angle45_offset7_P, Inertia_angle45_offset7_P, LongRunEmphasis_angle135_offset1_P, uniformity_P, Percentile5_P) and 9 features from CECT images (Correlation_AllDirection_offset7_SD_A, Correlation_angle90_offset1_A, GLCMEnergy_AllDirection_offset7_SD_A, GreyLevelNonuniformity_AllDirection_offset1_SD_A, HaralickCorrelation_AllDirection_offset1_SD_A, HighGreyLevelRunEmphasis_AllDirection_offset7_SD_A, HighIntensitySmallAreaEmphasis_A, kurtosis_A, ShortRunEmphasis_angle0_offset1_A). Logistic regression was used to construct the radiomics signature. The rad-scores were significantly different between the training group and the test group. The predictive effects of the two groups of patients were favorable, with AUCs of 0.772 and 0.771, specificities of 0.878 and 0.889, and sensitivities of 0.808 and 0.790, respectively (see **Figure 4** for details). Detailed information about the dimensionality reduction procedures and results can be found in the **Supplementary Materials**.

### Classification Model Construction and Validation

**Figure 5:**
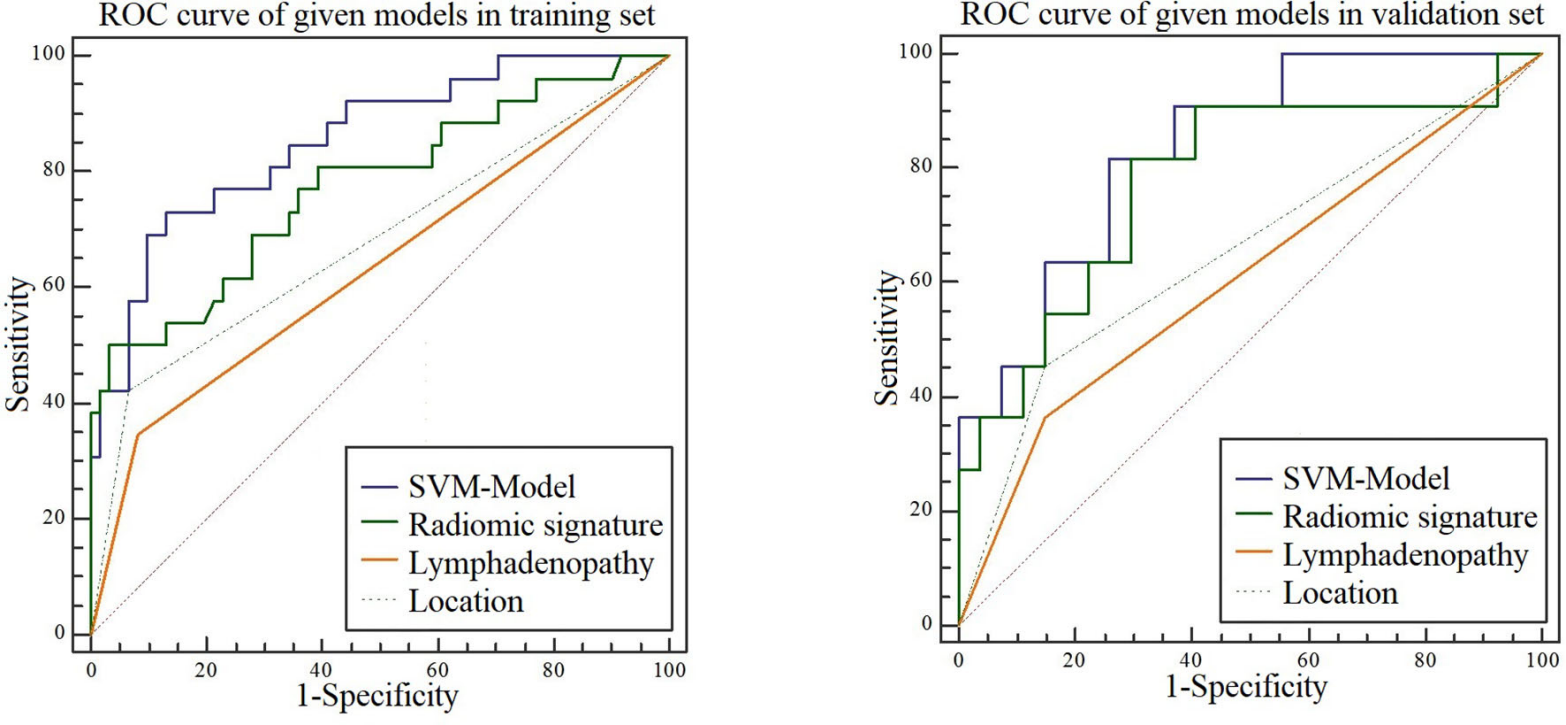
ROC curves for the SVM model, radiomic signature, location, and lymphadenopathy when predicting malignancy in the training and test sets.

Analysis of variance and multivariable logistic regression analysis showed that location, lymph node status, and rad-score were independent predictors of benign and malignant tumors. See **Table 4** for details. ROC curve analysis shows that the accuracy of the SVM-based prediction model, radiomics signature, location and lymph node status in the training set was 0.854, 0.772, 0.679, and 0.632, respectively; the specificity was 0.869, 0.878, 0.734, and 0.773; and the sensitivity was 0.731, 0.808, 0.723 and 0.742. In the test set, the accuracy was 0.835, 0.771, 0.653, and 0.608, respectively; the specificity was 0.741, 0.889, 0.852, and 0.812; and the sensitivity was 0.818, 0.790, 0.731, and 0.716. Details are shown in **Figure 5**.

**Table 4:** Multivariable logistic regression analysis for selecting predictors for model construction.

Variable	ANOVA		Multivariable logistic regression		
	F value	*P* value	OR (95%CI)	*P* value	VIF value
Sex	1.556	0.216			
Age	0.078	0.781			
Duration	0.018	0.893			
Smoking	1.89	0.173			
Distribution	0.812	0.37			
Location	19.64	<0001*	0.21(0.047–0.936)	0.041*	1.195
Maximum diameter	1.436	0.234			
Shape	0.101	0.752			
Capsule	3.513	0.064			
Regularity	1.926	0.169			
Border	5.874	0.017*	NA	NA	
Density	0.127	0.722			
Enhancement	0.098	0.755			
Marked Enhancement	1.086	0.3			
Cystic degeneration	0.093	0.761			
Lymphadenopathy	10.325	0.002*	4.694(1.06–20.789)	0.042*	1.062
Hemorrhage	2.384	0.126			
Calcification	0.798	0.374			
Heterogeneity	0.567	0.454			
Radiomics signature	29.375	<0.001*	2.521(1.432–4.438)	0.001*	1.18

NA, Not available. *represents P < 0.05.

**Figure 6:**
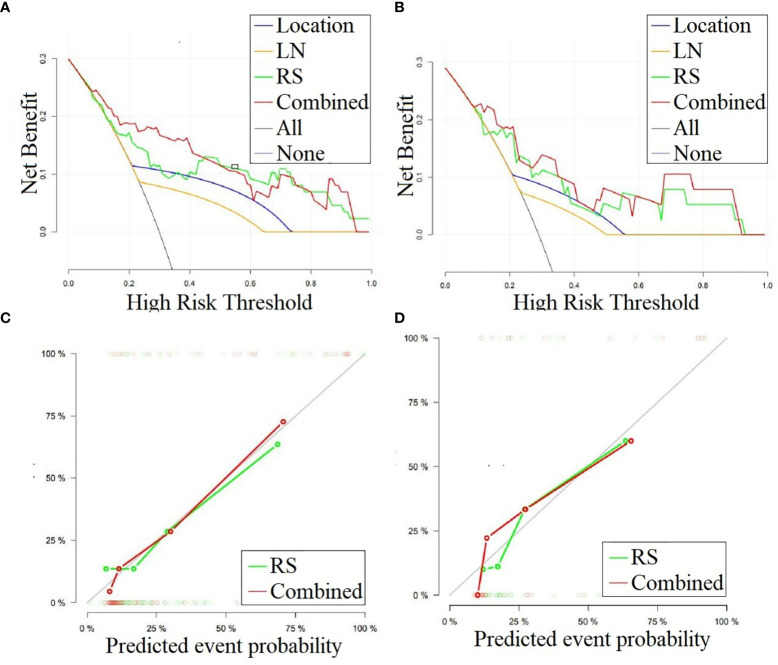
**(A, B)** DCA curves for the combined model, radiomics signature, lymphadenopathy and location in classifying parotid gland tumors in the training and test sets, respectively. The graphs show that the combined model has the greatest net benefit for both datasets. **(C, D)** Calibration curves for the combined model and radiomics signature in classifying the parotid gland tumors in the training and test sets, respectively. LN, Lymphadenopathy; RS, radiomic signature.

**Figure 7:**
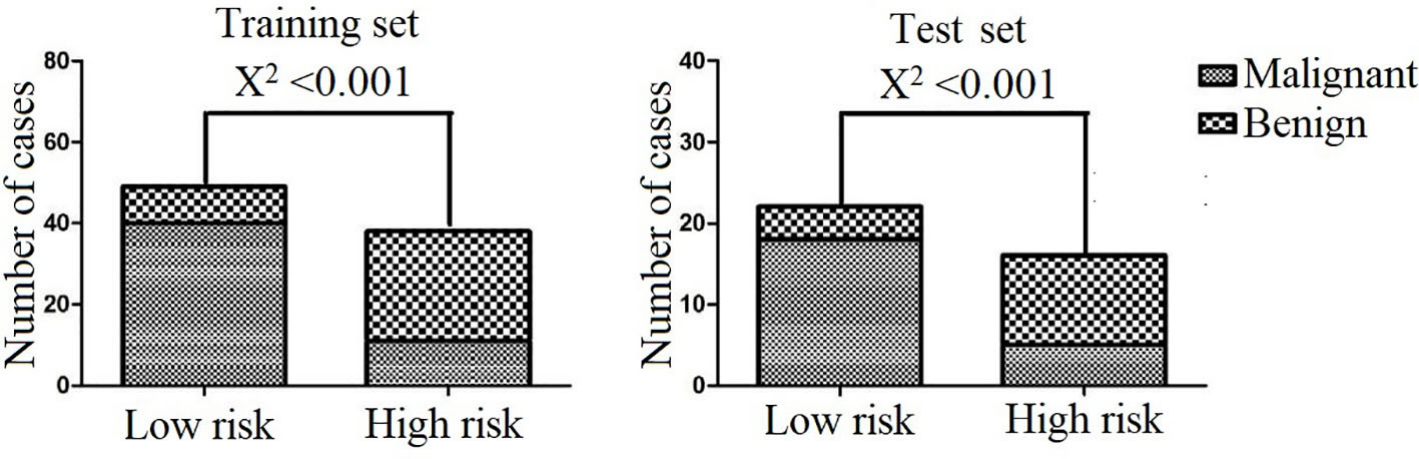
Classification performance of the combined model in the training and test sets.

We performed DCA for the SVM model, radiomics signature, location, and lymphadenopathy in the training and test sets. The results show that the SVM model has the greatest net benefit in both datasets. Additionally, we conducted calibration curve analysis of these continuous variables in both datasets, and they all showed good consistency, as shown in **Figure 6**. According to the optimal diagnostic cut-off value of the model (0.323), patients were divided into a low-risk group and a high-risk group. There were significant differences in the number of malignant PG tumors between the low-risk group and the high-risk group in both the training set and test set (P<0.0001), as shown in **Figure 7**.

## Discussion

We developed and validated a combined prediction model based on radiological data and CT-radiomics for differentiating benign and malignant PG tumors in two independent clinical cohorts. The combined model was constructed by incorporating the rad-score from the radiomics signature and two radiological features. The rad-score was calculated using the LR model, which was developed with 14 selective features, including five features from NCCT and nine features from CECT images of PG tumors. The combined SVM model outperformed the radiomics signature and individual radiological features in both the training and test groups. Thus, the proposed non-invasive method of the favorable, combined prediction model makes it a potential preoperative evaluation tool in clinical practice.

Medical imaging is one of the major factors in clinical evaluation and treatment. However, traditional medical imaging is primarily a subjective or qualitative science. Radiomics, a relatively newly developed set of techniques, allows the high-throughput extraction of imaging features to quantify the different characteristics that oncologic tissues exhibit in medical imaging (20). Recently, there have been several studies on the application of radiomics to PG disease. Ajmi et al. (16) used dual-energy CT to investigate the classification of two benign parotid tumors, Warthin tumors and pleomorphic adenomas. Pallamar et al. (21) utilized standard MRI sequence-based textures to discriminate PG masses; however, only a rather small 38 patients with various pathological entities were enrolled, and regions of interest derived from only three slices rather than from the whole tumor were used for extracting a limited number of features. Another study (22) used only the arterial phase to distinguish pleomorphic adenoma from Warthin tumor, both of which are benign tumors and have similar clinical management. In addition, several studies have investigated the changes in the parotid morphology and secretory function induced by radiotherapy for head and neck cancers (23, 24). Unlike the above-mentioned literature, we performed 3D whole tumor analysis to differentiate benign and malignant PG tumors with larger, multicentre datasets of both plain CT and contrast-enhanced CT images. Furthermore, machine learning methods were employed to ensure robustness and reproductivity, which make this study more clinically practical.

In this study, 14 texture features were selected with a machine learning method from both NCCT and CECT images to develop a radiomics signature, including first-order and high-order features. Previous literature has demonstrated that radiomics features may reflect relevant and potentially important phenotypic information, such as intra-tumor heterogeneity, subsequently providing valuable information for diagnosis, prognosis and individualized therapy (25). Our results showed that several GLCM features survived as robust in the radiomics signature and participated in the construction of the prediction model. GLCM features describe the relationship between two neighboring pixels, which could potentially reflect local intra-tumor heterogeneity and is associated with tumor malignancy. It is hypothesized that intra-tumor heterogeneity can be exhibited at several spatial levels—macroscopic, cellular and molecular (genetics)—, all leading to radiological differences; thus, radiological tumor phenotype characteristics may be useful for investigating the underlying evolving biology and have been reported to be associated with worse survival in tumor patients (26). Our radiomics result was consistent with that of Zhang (27), who found that texture features, mainly consisting of GLCM features, in malignant PG tumors were significantly different from those in benign tumors. It can be surmised that malignant tumors grow rapidly and are mostly infiltrating, resulting in insufficient blood supply, which can easily cause microbleeds and necrosis of the tumor. Therefore, the heterogeneity of malignant lesions is higher than that of benign lesions.

Our study employed both plain CT and CECT to extract features to construct the radiomics signature as PG tumors have different CT densities for different tumor histologies and are mainly supplied by arterial blood, yielding more texture information than only the arterial phase, as used in a previous study (22). More features were selected from CECT images than from NCCT images; thus, CECT may reflect more important information, and it may be speculated that unlike plain CT, CECT might also reflect some heterogeneous features associated with the tumor blood supply (3). Our results may further suggest that blood supply information is different between benign and malignant PG tumors, which may be because tumor capillaries generally have wider inter-endothelial junctions and a large number of discontinuous or absent basement membranes in malignant tumors, which result in different haemodynamic conditions in the arterial stage between benign and malignant tumors (28).

Our integrated model showed the best performance, followed by the radiomics signature alone and then individual radiological features, which indicates that although the radiomics features of the tumor itself had better predictive ability than the radiological features themselves, extra-tumor radiological information such as lymphadenopathy is also important; only by combining these two complementary features could the model provide a precise evaluation of the entire tumor for management.

Our study still has several limitations. First, the sample size was relatively small, with 87 patients in the training group. However, 38 patients were enrolled from another independent medical center as the test group to investigate the models’ reproducibility, and our results showed that the prediction model based on the training set was also stable for the test set. In the future, we will carry out further multicentre studies with a larger sample size. Second, the manual process of tumor segmentation and the reproducibility of radiomics features is one debatable aspect in radiomics analysis, as there is some subjectivity involved in the delineation of tumor boundaries. However, a recent study on the robustness and reproducibility of radiomics features suggested that only those reproducible features should be selected in building a radiomics model (29), which was employed in this study to ascertain the robustness of the features extracted from the segmented tumors by the two radiologists independently. In addition, ultrasound is sufficient for most benign tumors for primary diagnosis. However, CT can be used as a complementary assessment tool in some cases such as deep tissue involvement, recurrence, suspicious malignancy or large tumors.

## Conclusion

This study developed and validated a combined prediction model based on radiological data and CT radiomics features to distinguish benign and malignant PG tumors in two independent clinical cohorts; this model showed better prediction accuracy than the radiomics signature and radiological features alone. Thus, the proposed model could be used as a noninvasive prognostic or predictive biomarker for personal evaluation and could help clinicians guide surgical decisions. Multicentre and prospective validation studies with larger datasets should be further implemented prior to practical application of the model in the clinic.

## Data Availability Statement

The original contributions presented in the study are included in the article/**Supplementary Material**. Further inquiries can be directed to the corresponding authors.

## Ethics Statement

The studies involving human participants were reviewed and approved by the Zhejiang Provincial People’s Hospital. Written informed consent for participation was not required for this study is in accordance with the national legislation and the institutional requirements.

## Author Contributions

The study concept and design were carried out by YX, ZS, and XW. Literature research was collected by YX and YL. Clinical studies were conducted by YX, GS, and YL. Data and statistical analyses were performed by YX, PP, and ZS. XG and XW guarantee the integrity of the entire research study. All authors contributed to the article and approved the submitted version.

## Funding

This work was supported by the Fund of Health Commission of Zhejiang Province (2017KY230、2020KY402).

## Conflict of Interest

PP was employed by GE Healthcare.

The remaining author declares that the research was conducted in the absence of any commercial or financial relationships that could be construed as a potential conflict of interest.
