# Supplementary material for: The Role of Preoperative Computed Tomography Radiomics in Distinguishing Benign and Malignant Tumors of the Parotid Gland
Source: Front Oncol. 2021 Mar 10;11:634452. doi: 10.3389/fonc.2021.634452 (PMC7988088; doi:10.3389/fonc.2021.634452)
Supplement: Supplementary file 1 [file DataSheet_1.docx]

**The role of preoperative CT radiomics in distinguishing benign and malignant tumours of the parotid gland: a clinical multicentre study**

Yu-Yun Xu^1, #^, Zhen-Yu Shu^1, #^, Ge Song^2^, Yi-Jun Liu^1^, Pei-Pei Pang^3^, Xue-hua Wen^1*^, Xiang-Yang Gong^1,4*^

^1^ Department of Radiology, Zhejiang Provincial People’s Hospital, Affiliated People’s Hospital of Hangzhou Medical College, Hangzhou, China

^2^ Department of Radiology, Zhejiang Cancer Hospital, Hangzhou, China

^3^ GE Healthcare China, Shanghai, China

^4^ Institute of Artificial Intelligence and Remote Imaging, Hangzhou Medical College, Hangzhou, China

^#^These authors contributed equally to this work.

**^*^Corresponding to:** Xue-hua Wen (xuehuasuqian@126.com) and Xiang-Yang Gong (cjr.gxy@hotmail.com)

**ORCID:**

Yu-Yun Xu: 0000-0003-2818-852X

Zhen-Yu Shu: 0000-0002-4372-7897

Xue-hua Wen: 0000-0002-5504-0400

**1. Standardization of data**

Extracted texture features were standardized, which removed the unit limits of the data of each feature and converted it into a dimensionless pure value. This allowed the indexes of different units or orders to be compared and weighted. We used a z-score normalization to make the image intensities fit a standard normal distribution with $\mu\text{=0}$ and $\sigma\text{=1}$, where $\mu$ is the mean value of the images, and $\sigma$ is the standard deviation. The normalized values (also called z-scores) of the image intensities (*x*) were calculated as follows:

$$z=\frac{x-\mu}{\sigma}$$

After image z-score normalization, the number of radiomics features arrived at 378 from every ROI according to IPM software. Radiomics features included the histogram (42 features), Haralick (10 features), Formfactor (9 features), Gray-Level Co-occurrence Matrix (126 feature, GLCM), Run length matrix (180 features, RLM) and Gray Level Size Zone Matrix(11 features, GLSZM). The feature details are described in the table below.


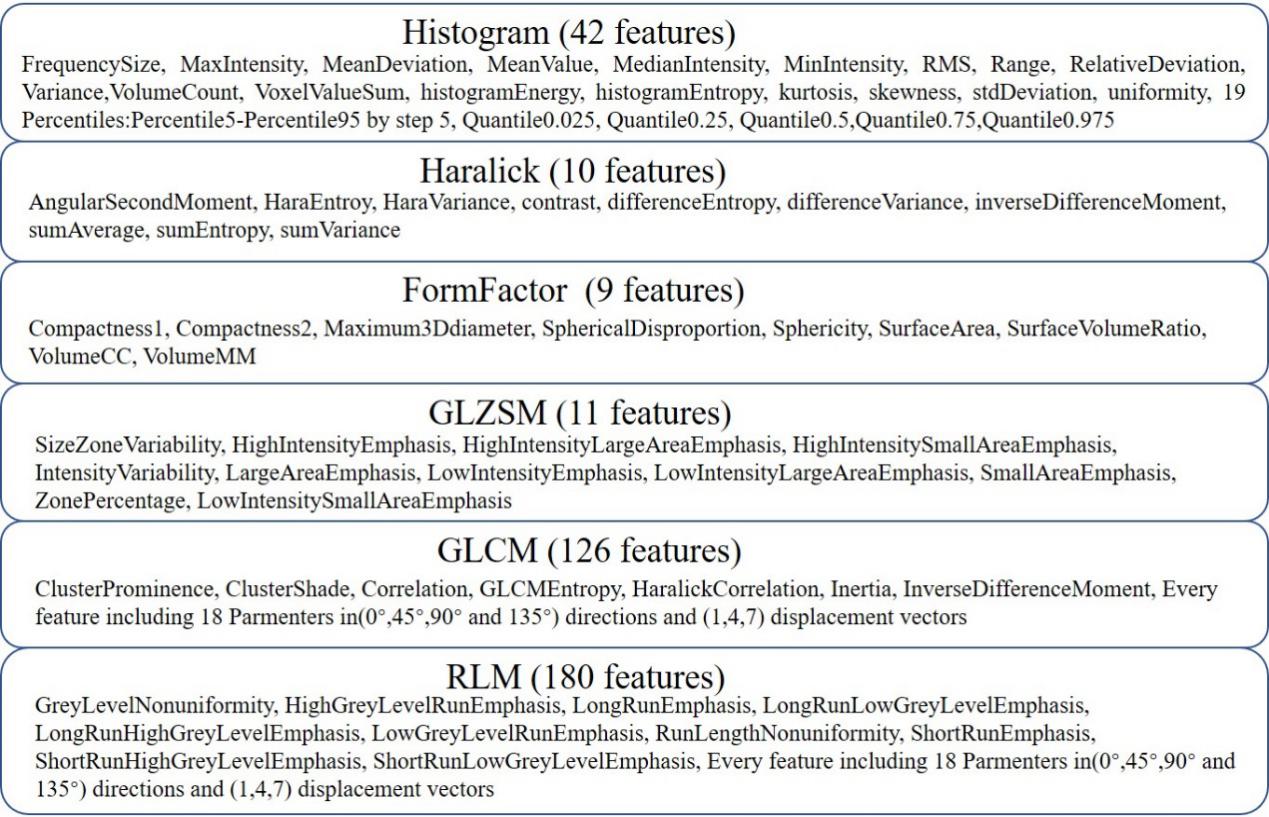


1. **Details on Dimension reduction**

After extracting a total 756 features from each patient’s CT data from NCCT and CECT, we first performed variance analysis on the radiomic features with 406 features left. Secondly, the mRMR algorithm was used to select the most powerful features, correlation analysis was performed on the remaining features after variance analysis using the minimal residual method to obtain 210 features, 91 features from NCCT and 119 features from CECT images. Then, from the minimal residual features, 41 were selected as the most relevant feature with the classification, 20 features from NCCT and 21 features from CECT images. Finally, the gradient boosting decision tree (GBDT) algorithm was used to reduce the dimension of the remaining features. GBDT is an algorithm that classifies or regresses data by the linear combination of basic functions and reduces the Residual generated in the training process. In this study, the GBDT algorithm was used to obtain 14 features, 5 features from NCCT and 9 features from CECT images. In addition, we performed a logistic regression analysis of these remaining features and a radiomic signature was built.

Detailed features are as follows:

'Correlation_angle90_offset1_A'

'Percentile5_P'

'uniformity_P'

'HighIntensitySmallAreaEmphasis_A'

'Correlation_AllDirection_offset7_SD_A'

'GLCMEnergy_AllDirection_offset7_SD_A'

'GreyLevelNonuniformity_AllDirection_offset1_SD_A'

'HaralickCorrelation_angle45_offset7_P'

'HighGreyLevelRunEmphasis_AllDirection_offset7_SD_A'

'Inertia_angle45_offset7_P' 'kurtosis_A'

'ShortRunEmphasis_angle0_offset1_A'

'HaralickCorrelation_AllDirection_offset1_SD_A'

The publicity of the signature is as follows:

Rad-score=-1.00633834*Correlation_angle90_offset1_A+ 0.62886578* Percentile5_P -0.71686472*uniformity-0.65614971*HighIntensitySmallAreaEmphasis_A+ 0.11415375*LCMEnergy_AllDirection_offset7_SD_A+0.62744327*Correlation_AllDirection_offset7_SD_A +0.27512809*GLCMEnergy_AllDirection_offset7_SD_A -0.7651682*GreyLevelNonuniformity_AllDirection_offset1_SD_A-0.30312664* HaralickCorrelation_angle45_offset7_P+0.21255701*HighGreyLevelRunEmphasis_AllDirection_offset7_SD_A -0.1267436* Inertia_angle45_offset7_P + 0.29447408* kurtosis_A+0.08693406*ShortRunEmphasis_angle0_offset1_A-0.02808784*aralickCorrelation_AllDirection_offset1_SD_A

3. The definition of the radiological features

All original CT images were reviewed and assessed by two experienced head and neck radiologists who were blinded to the clinical data. If there were multiple lesions in the parotid gland, the largest lesion with confirmed pathology was chosen for the analysis. The definitions of the imaging characteristics are listed as follows:

Location: The lobes are divided by the retromandibular vein into shallow and deep lobes. Thus, the location of the tumor was defined as in shallow lobe if it is exterior to the retromandibular vein, or in deep lobe interior to the retromandibular vein.

Maximum diameter: The sizes of the tumors were measured by determining the maximal cross-sectional diameter.

Lymph node metastasis (with or without): We assessed the node metasatasis based on the imaging characteristics, including clustering, rounded shape, inhomogeneity, size, periphery invasion, sentinel location. The maximal axial dimension criteria for metastatic lymph nodes are >15 mm for level I and II nodes, 8 mm for retropharyngeal nodes and 10 mm for all other node levels.

Distribution: When there is more than one mass in the parotid gland, if the tumor is in one parotid gland, it is defined as unilateral, otherwise it is considered bilateral.

Enhancement: We measured CT attenuation values (in HU) on plain CT and enhanced CT scans by placing the largest possible circular region of interest within the solid portion of the lesion with caution to avoid the cystic area. Then, the tumors were defined as enhanced if the attenuation of postcontrast CT scan was 20 Hounsfield units (HUs) more than the precontrast CT scan.

Cystic degeneration: cystic area was defined as having a CT scan attenuation of 25 HU or less.

Density: The CT density of the parotid gland was used as a reference, tumors with a CT value equal to or higher than the CT value of the parotid gland are considered as iso-hyperdense, and tumors with a CT value lower than the parotid gland's CT value are considered hypodense.

Enhancement type: Marked enhancement is defined as the CT value of tumor enhancement on postcontrast CT is 40Hu higher than it on plain CT scan.

Calcification: Calcification is defined as the CT value of the foci within the tumor is higher than 100Hu.

4. The demographics between the included and excluded groups were compared, including the age, sex, smoking habit and duration of the disease.

Table 1. Clinical characteristics of patients in the included and excluded sets.

| Variable | | Included set (n=125) | Excluded set (n = 83) | *P* value |
| --- | --- | --- | --- | --- |
| Age (years) | | 53.34±16.23 | 52.21±16.39 | 0.626 |
| Duration (months) | | 28.65±58.43 | 32.87±63.09 | 0.622 |
| Sex [n (%)] | Male | 62(49.6) | 44(53) | 0.63 |
|  | Female | 63(50.4) | 39(47) |  |
| Smoking [n (%)] | Yes | 31(24.8) | 19(22.9) | 0.752 |
|  | No | 94(75.2) | 64(77.1) |  |
| Malignancy [n (%)] | Yes | 37(29.6) | 27(32.5) | 0.654 |
|  | No | 88(70.4) | 56(67.5) |  |
